# *matK*-QR classifier: a patterns based approach for plant species identification

**DOI:** 10.1186/s13040-016-0120-6

**Published:** 2016-12-09

**Authors:** Ravi Prabhakar More, Rupali Chandrashekhar Mane, Hemant J. Purohit

**Affiliations:** 1 Environmental Genomics Division, CSIR-National Environmental Engineering Research Institute, Nagpur, 440020 Maharashtra India; 2 MDS Bio-Analytics, Nagpur, 440020 Maharashtra India; 3 Present Institute: Division of Molecular Entomology, ICAR- National Bureau of Agricultural Insect Resources (NBAIR), Hebbal, Bengaluru, 560024 Karnataka India

**Keywords:** Plant species identification, *MatK* and *rbcL* loci, Regular expression, Signatures, *MatK* classifier software

## Abstract

**Background:**

DNA barcoding is widely used and most efficient approach that facilitates rapid and accurate identification of plant species based on the short standardized segment of the genome. The nucleotide sequences of maturaseK (*matK*) and ribulose-1, 5-bisphosphate carboxylase (*rbcL*) marker loci are commonly used in plant species identification. Here, we present a new and highly efficient approach for identifying a unique set of discriminating nucleotide patterns to generate a signature (i.e. regular expression) for plant species identification.

**Methods:**

In order to generate molecular signatures, we used *matK* and *rbcL* loci datasets, which encompass 125 plant species in 52 genera reported by the CBOL plant working group. Initially, we performed Multiple Sequence Alignment (MSA) of all species followed by Position Specific Scoring Matrix (PSSM) for both loci to achieve a percentage of discrimination among species. Further, we detected Discriminating Patterns (DP) at genus and species level using PSSM for the *matK* dataset. Combining DP and consecutive pattern distances, we generated molecular signatures for each species. Finally, we performed a comparative assessment of these signatures with the existing methods including BLASTn, Support Vector Machines (SVM), Jrip-RIPPER, J48 (C4.5 algorithm), and the Naïve Bayes (NB) methods against NCBI-GenBank *matK* dataset.

**Results:**

Due to the higher discrimination success obtained with the *matK* as compared to the *rbcL*, we selected *matK* gene for signature generation. We generated signatures for 60 species based on identified discriminating patterns at genus and species level. Our comparative assessment results suggest that a total of 46 out of 60 species could be correctly identified using generated signatures, followed by BLASTn (34 species), SVM (18 species), C4.5 (7 species), NB (4 species) and RIPPER (3 species) methods As a final outcome of this study, we converted signatures into QR codes and developed a software *matK*-QR Classifier (http://www.neeri.res.in/matk_classifier/index.htm), which search signatures in the query *matK* gene sequences and predict corresponding plant species.

**Conclusions:**

This novel approach of employing pattern-based signatures opens new avenues for the classification of species. In addition to existing methods, we believe that *matK*-QR Classifier would be a valuable tool for molecular taxonomists enabling precise identification of plant species.

**Electronic supplementary material:**

The online version of this article (doi:10.1186/s13040-016-0120-6) contains supplementary material, which is available to authorized users.

## Background

In recent years, DNA barcoding is considered as a universal species identification method for plants. It mainly involves discrimination of species through standardized molecular marker gene and is gaining support from the taxonomists as well. DNA barcoding has wider applications in different studies namely to predict cryptic species, to study biological samples in forensics and conservation sciences for characterization of biodiversity; to track inventory for plants identity or purity and in ecological species diversity studies [1–4]. Various molecular markers have been used for DNA barcoding studies. A 650 base pair (bp) of the mitochondrial cytochrome c oxidase unit I (*COI*) gene was used as a barcode in various organisms such as animals, birds, fishes, insects and nematodes [4–8]. A specific region of the nuclear ribosomal internal transcribed spacer (*ITS*) gene is the well-studied DNA barcode for fungi [9]. In plant DNA barcoding, there has been extensive debate about the locus choice; several regions of the genome (*trnH, psbA, rpoC1, rpoB, atpF, atpH, psbK,* and *psbI*) were referred as candidate markers with different discrimination success. However, the two loci ribulose-1,5-bisphosphate Carboxylase (*rbcL*) and maturase K (*matK*) gene regions are widely used in plant barcoding studies for phylogenetic analyses or species identification [10, 11]. Seven chloroplast loci have been tested for plant species identification by The Consortium for the Barcode of Life (CBOL) Plant Working Group, where the suitability of *matK* and *rbcL* loci as a barcode was showed [12]. Vinitha et al. [13] studied a total of 20 species belonging to the family Zingiberaceae from India by using nine plastids and two nuclear loci and reported that *matK* and *rbcL* aids in the determination of 15 species (75%) into monophyletic groups. Techen et al. [14] mentioned that the *matK* region was preferred as a barcode candidate because of high evolutionary rate, low transition/transversion rate, and inter-specific divergence.

In plant DNA barcoding, data mining of marker genes is a noteworthy step that directly assists in species identification. Towards practical uses of DNA barcoding in order to assign sequences to species, various methods have been proposed. The tree-based phylogenetic analysis is a popular method for estimate time of divergence between a group of organisms and relationship among the species. The most common method for species identification is Basic Local Alignment Search Tool (BLAST) followed by distance matrix computations. There could be a possibility that the phylogenetic tree-based method (like Neighbor-joining (NJ) or Maximum Likelihood) gives the lowest accuracy due to unavailability of homologs in databases [15]. To identify species, the TaxI program was developed using distance models to compute the sequence divergences between a query and reference sequences [16]. Diazgranados and Funk [17] published a Quick Response (QR) barcoding system on specimens for biological collections. On a similar line, QR code symbols were implemented to encrypt the five different loci sequences by Liu et al. [18] and performed the species identification based on combined BLAST and distance-based methods.

Weitschek et al. [19] evaluated successfully the performance of the function-based method i.e. Support Vector Machines (SVM), Naïve Bayes, the rule-based RIPPER, and the decision tree C4.5 for DNA barcodes classification purposes, but their performance was not consistent with all taxonomic studies. The exact taxonomic classification of barcodes with highly similar and closely related taxa sequences remains problematic due to the algorithmic bias. Although *matK* and *rbcL* loci are used for most of the studies, but it has been found that they are having variable regions with few base pairs. As more plant DNA barcodes based on multiple loci became available, there will be an inclination towards the study of gene-specific species identification. In a recent study, we uncovered a role for discriminating patterns in 16S rRNA gene sequences and generated four taxa signatures (i.e. *Bacilli, Bacillales, Bacillaceae,* and *Bacillus*) for taxonomic classification through alignment-free DNABarID software in Bacteria [20]. Similarly, Weitschek et al. described the use of logic alignment-free approach for bacterial genome identification [21]. However, there is no single software available that uses gene-specific (*matK*) multiple nucleotide patterns for plant species identification. Therefore, one ongoing challenge for DNA barcoding is to develop a precise and reliable approach for speed up species identification process. It is important to note that marker genes hold the discriminating and unique nucleotide patterns which are mainly responsible for sequence classification. In view of this, Position Specific Scoring Matrix (PSSM) could be implemented to detect variable sites in the gene which can further be used for species identification. PSSM is an alignment based method commonly used for a pattern or motif discovery in nucleotide and amino acid sequences and tested to analyze *matK* gene sequences of *Nymphaea* and *Nuphur* plants of basal angiosperms [22]. However, characters-based PSSM approach is also being used to identify species and is often more effective for analyzing nucleotide sequences [23, 24]. Here we present a signatures-based search method and the corresponding software tool, *matK*-QR Classifier on the Windows platform (http://www.neeri.res.in/matk_classifier/index.htm), which is different from the early tools in the set of predictive nucleotide patterns and the method of sequence classification. Molecular signatures (here we referred as a regular expression) are defined as DNA nucleotide motifs that are unique and present in target species but different from other species sequences [25].

In the present study, we try to show the pattern based utility for species identification by targeting *matK* gene using PSSM method. Similar approaches can be carried out in order to study plant DNA barcodes studies on individual or combined loci, where existing methods are not sufficient to discriminate species. Rubino and Attimonelli [26] described the use of Regular Expression Blasting algorithm using the information of multiple aligned sequences. In order to generate regular expression or signatures for species identification, it is important to find the discriminating nucleotide patterns and their precise positions in the marker gene. To meet this goal, we assessed the discrimination power of *matK* and *rbcL* loci datasets of the CBOL Plant Working group [12]. We obtained the discriminating patterns of *matK* gene sequences at genus and species levels and generated 60 species signatures by using the MSA and PSSM data. We encrypted the signatures into the QR code for graphical representation. Additionally, we performed a comparative assessment of signatures with other sequence classification software’s and showed the suitability of the generated signatures in terms of sensitivity and specificity for plant species identification.

## Methods

**Fig. 1:**
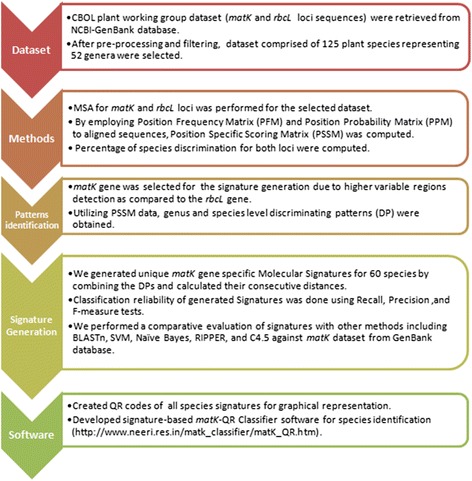
The overall workflow used for pattern-based signatures generation in the present study

In this section, we described a workflow aspects geared toward methodological signature generation, which includes the methods regarding developing a strategy for signature, use of the statistical method, signature evaluation, and software implementation. The species signature generation consisting of data retrieval, analysis by various methods, search for various patterns and signatures and finally the creation of software. The overall workflow used for present study is illustrated in Fig. 1. All analysis were performed using Perl (BioPerl 1.6.1), R (2.15.2) statistical programming scripts and partly with text editor EditPadPro 7.2.3 (www.editpadpro.com) on a workstation (Intel Xeon CPU, 2.80GHz (2 processors), 12 GB RAM).

### Retrieval of dataset for signature generation

We referred the plant species barcode dataset reported by the CBOL Plant Working Group and Janzen DH for the present study, which is available at GenBank Nucleotide Database [12]. The dataset comprised of total 404 species (mainly from angiosperms, cryptogams, and gymnosperms) representing seven candidate plastid DNA barcoding loci (*rpoC1, rpoB, matK, trnH-psbA, rbcL, atpF-atpH,* and *psbK-psbI*). The dataset used for downstream analysis have been chosen with the following filters: (i) sequences with *matK* and *rbcL* loci; (ii) species with two or more representative sequences; and (iii) excluded species with N’s in the sequences. After filtering, the final dataset comprises 344 DNA Barcode sequences from 125 species, which represents a total of 52 genera. The details of two selected loci datasets are summarized in Additional file 1.

### Position specific scoring matrix (PSSM) analysis

We performed multiple sequence alignment (MSA) of *matK* and *rbcL* data sets using ClustalW2 software implemented in BioEdit 7.2.5 [27]. Then, the gap region in alignments was trimmed and obtained equal length alignment file for downstream analysis. The distance matrix was created to estimate the evolutionary distances using PSSM in R statistical package. PSSM method includes the relative frequencies obtained by counting the occurrence of each nucleotide at each position of the alignment, followed by normalization of the frequencies. Thus, for a set X with N aligned sequences of alignment length c, the elements of PSSM were calculated using the equation below:

$$ {M}_{k,j}=\frac{1}{N}{\displaystyle \sum_{i=1}^NI\left({X}_{i,j}=k\right)} $$

Where, *i* = 1, 2, …, *N* refers to aligned sequence; *j* = 1, 2, …, *c* refers to a column position of alignment; I is an indicator function taking value either 0 or 1; *k* is the set of 4 nucleotides (i.e. A, T, G, and C).

PSSM considers statistical independence among the adjacent positions, as the probabilities for each position are estimated independently of other positions. We considered the sum of the values in a particular position equals 1.0. Each column of the alignment can be viewed as an independent multinomial distribution. To determine the distance between two sequences, the probability of a nucleotide at each position in each sequence was generated. The Euclidean distance between the two probability arrays was calculated as a measure of similarity between the two sequences.

### The percentage of species discrimination analysis

To assess the discriminatory power of *rbcL* and *matK* loci, the pairwise sequence divergence was calculated using PSSM information and subjected to the percentage of species discrimination. For comparing the sequence variation at different loci, Wilcoxon signed-rank test was used. The success rate of species discrimination is the proportion of species that could be differentiated from all possible species pairs. A pair of species was scored as successfully discriminated if the inter-species distance was higher than the intra-species distance and zero. Finally, we obtained a total number of species by using *matK* and *rbcL* loci. All analysis steps were performed with R statistical package.

### Identification of discriminating patterns

We performed two-step (genus and species level) analysis to get the discriminatory patterns in the *matK* gene. In the first step, at the genus level, we grouped the genera wise sequences and performed MSA to get the conserved consensus sequence for each genus. We computed Position Frequency Matrix (PFM) and Position Probability Matrix (PPM) and obtained the variable sites as discriminating patterns among the genus consensus sequences. In the subsequent step, at the species level, we performed MSA of all sequences from each genus to obtained discriminating patterns.

### Species signature generation

To generate the signature (i.e. regular expression), we created two datasets (i.e. target species and other species). After discriminating pattern identification, the signature generation process was carried out by the following main steps: the identified position-specific discriminating nucleotides were marked as one in target species and were considered as discriminating patterns; next, we determined the nucleotide bp distances between two consecutive patterns in target species sequences. The patterns and their ordered arrangement with distances in target species were taken into account for signature generation. Additionally, we referred to the MSA for identification of single bp change among the species. We incorporated few nucleotides into the signatures which can discriminate against false positive hits. All the species signatures were generated by performing the same procedure and searched against the target and other species sequences. We determined the recall and precision for each signature as:

- Recall = True Positives/(True Positives + False Negatives),
- Precision = True Positives/(True Positives + True Positives), and
- F-measure = 2((Precision*Recall)/(Precision + Recall)).

Among the different possible signatures for a species, one with higher sensitivity and specificity was selected as the candidate signature for that species.

### Comparative assessment of signatures with other methods

To evaluate all the signatures with sequences present in public database, we retrieved *matK* sequence data set from the NCBI-GenBank database, with the following filters (Species: plants, Source database: INSDC GenBank and RefSeq, Genetic Component: Chloroplast, Sequence length: <2000). The data comprises 89890 *matK* gene sequences. The sensitivity and specificity of each species signature were tested against the data set.

For comparative assessment of signatures with other methods, we have sub-sampled all the 60 species sequences from the *matK* dataset. The final data set comprises 442 query sequences. We evaluated classification performance of signatures with homology-based BLASTn, the function-based method Support Vector Machines (SVM), Naïve Bayes, the rule-based RIPPER, and the decision tree C4.5 using WEKA data mining tool. We converted *matK* FASTA sequences to the WEKA format and used the procedure published by Weitschek et al. [19] for DNA barcode sequence classification using the WEKA tool. We performed similarity search of all query sequences against the GenBank nucleotide (nt) database as on 28-02-2016, using standalone BLASTn 2.2.24 [28]. The species name of the first hit was assigned to each of the query sequences. To test the performance of species identification accuracy, obtained hits were used to determine the recall, precision, and F-measure for all the methods.

### QR code signature based ‘matK-QR Classifier’ software

We developed ‘matK-QR Classifier’ software for species identification through the generated 60 plant species signatures. For symbolic representation, we encrypted each signature into QR code using Free QR Creator software (http://www.smp-soft.com). This software performs regular expression based search between species signatures (QR-coded) against a query *matK* gene sequences and assigns species if the signature is matched. This software is based on Windows platform. Perl script was written for regular expression search, which was converted to the executable file using the software Perl Dev Kit (PDK) (www.activestate.com/perl-dev-kit). The front-end design of the software was implemented in C# programming language.

## Results

### Alignment, PSSM and species discrimination

Multiple sequence alignment of the *matK* and *rbcL* loci datasets (125 species) produced a final of 520 and 532 bp aligned regions respectively. To further extend our analysis, we generated the position specific scoring matrix from each alignment and created a conserved matrix of the four nucleotides (i.e. A, T, G, and C). Proportional conservation at each position was calculated ranging from 0 to 1. For instance, if G is completely conserved at the position, then the estimated value is 1. The generated PSSM information comprised a positional frequency matrix (PFM) and positional probability matrix (PPM) for both loci sequences (Additional file 2). Based on PSSM information, the pairwise distances between all species sequences were computed and a distance matrix was created for species discrimination analysis.

Species discrimination results for both loci using PSSM method are given in Table 1. The result revealed that a total of 75 and 71 out of 125 species were discriminated for *matK* and *rbcL* respectively. However, remaining species could not be distinguished due to the lack of variable sites in sequences. The discriminated and non-discriminated species names are listed in Additional file 3.

**Table 1:** The discriminating and not discriminating species in *matK* and *rbcL* loci using PSSM method

No.	Locus	No. of discriminating species	No. of non-discriminating species
1	*matK*	75	50
2	*rbcL*	71	54

### Identification of discriminating patterns and species signature generation

Due to the higher discrimination success given by the *matK* as compared to the *rbcL*, we selected *matK* gene for signature generation. The variable nucleotide sites (i.e. discriminating patterns) for each genus are listed in Additional file 4.

Finally, the genus and species level discriminating patterns were combined and considered for signature generation. Species signatures generated were unique in terms of a combination of species-specific nucleotide patterns and the exact distance between two consecutive patterns.

**Fig. 2:**
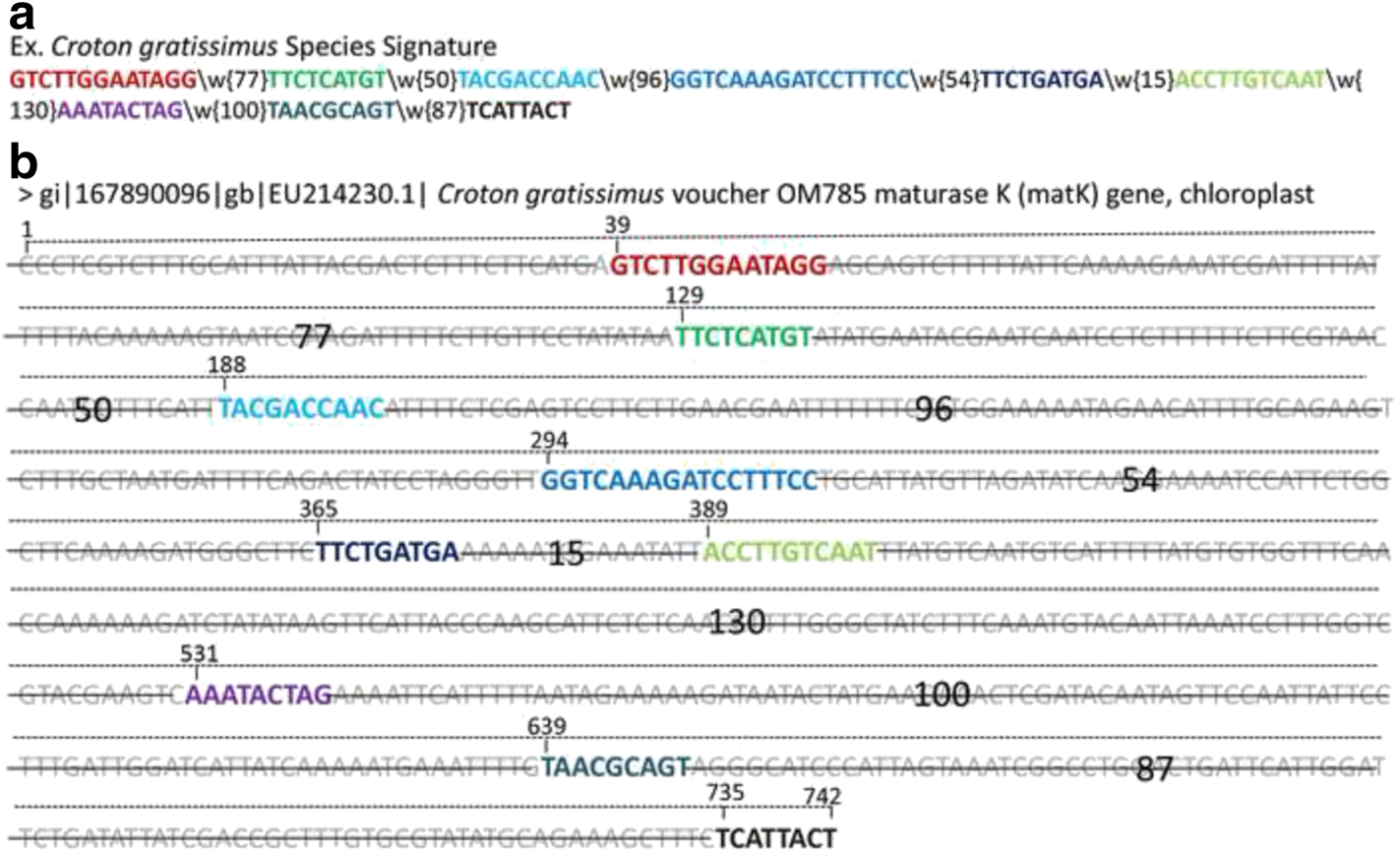
An example of the representation of the species signature in nucleotide maturase K (*matK*) gene sequence (**a**) *Croton gratissimus* (**b**) *Croton gratissimus* voucher OM785 (EU214230.1). The patterns are highlighted in the same color in signature and sequences

By performing the sensitivity and specificity test for all the signatures, we generated the signatures for 60 out of the 75 species (Additional file 5). We could not generate signatures for the remaining 15 species due to the mismatch of nucleotides distances. Different signatures have been covered different spanning region in the *matK* gene. This is due to the fact of a different number of patterns, size, and base pair distances between consecutive patterns. For instance, the *Croton gratissimus* species signature is given in Fig. 2a. All patterns of the signature are mapped on one representative *matK* gene sequence having GenBank accession number EU214230.1 (Fig. 2b). The signature comprised of total 9 discriminating patterns, which are spanning a 704 bp region from nucleotide position 39 to 742 bp in total sequence length of 742 bp. The unique discriminating patterns are present at 39, 129, 188, 294, 365, 531, 639 and 735 start positions in the sequence, which make signature unique to this particular species.

### Nucleotide patterns evolution among the species

**Fig. 3:**
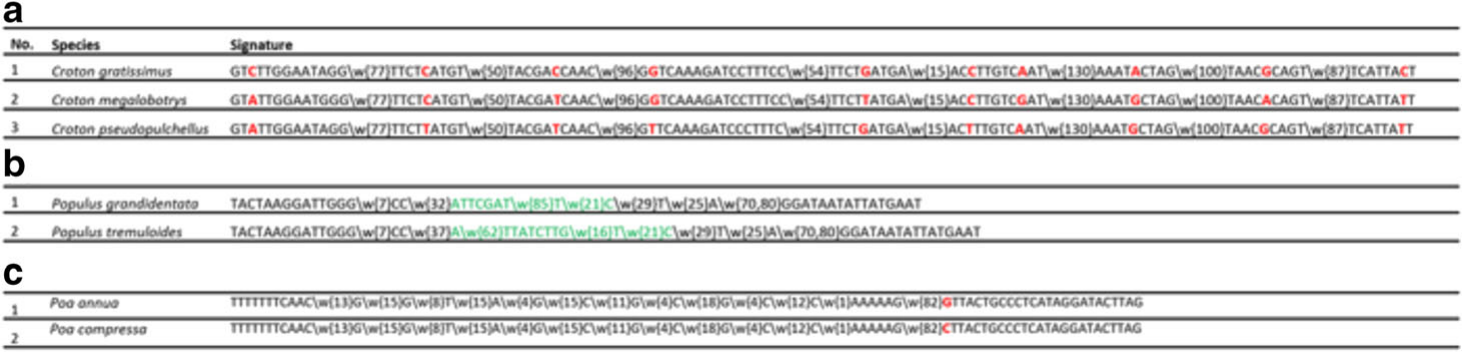
Evolution of nucleotide patterns of signatures in species of the genus (**a**) *Croton* (**b**) *Populus* (**c**) *Poa.* The key discriminating nucleotide positions are highlighted in *red*

To understand the evolution of patterns among the species and its role in sequence discrimination, we described species signatures of three genera, which are representing three different scenarios (Fig. 3). For instance, the species signatures (for *Croton gratissimus, Croton megalobotrys,* and *Croton pseudopulchellus)* of the genus *Croton* are displayed in Fig. 3a. As apparent from Figure, though the nucleotides distance among the patterns is similar in all the three signatures, a single nucleotide substitution in all the nine patterns is found to be responsible for inter-species discrimination. Among the signatures of *C. gratissimus* and *C. megalobotry*, the third position in the first pattern (GTCTTGGAATAGG) has been substituted (C/A). However, this substitution is not observed among *C. megalobotry* and *C. pseudopulchellus.* Similarly, among the signatures of *C. megalobotry* and *C. pseudopulchellus*, the fifth position in the second pattern (TTCTCATGT) has been substituted (C/T) but not between *C. gratissimus* and *C. megalobotry*. This shows that dissimilar patterns are responsible for the closest species discrimination. For the genus *Populus* (Fig. 3b), the distinctive patterns (highlighted in green) along with its bp distances are crucial factors for discrimination of the two species. In the case of the genus *Poa* (Fig. 3c), it can be observed that a single bp difference (G/C) in the last pattern (highlighted in red) play a significant role in the classification of *Poa annua* and *Poa compressa* species.

### Signatures evaluation and comparative assessment of other methods

We evaluated the sequence classification performance of 60 species signatures against the *matK* sequences in the NCBI-GenBank database. For comparative assessment purpose, we compared generated signatures with the BLASTn, SVM, Naïve Bayes, RIPPER, and C4.5 methods. Recall and precision are usual criteria for identifying the accuracy level of different species identification methods, and the F-measure is one of the important evaluation features which can be applied for selecting the best classification method. To get detailed information about how the sequences are assigned to the same and other species among different algorithms, we determined the recall, precision, and F-measure for all the methods.

**Fig. 4:**
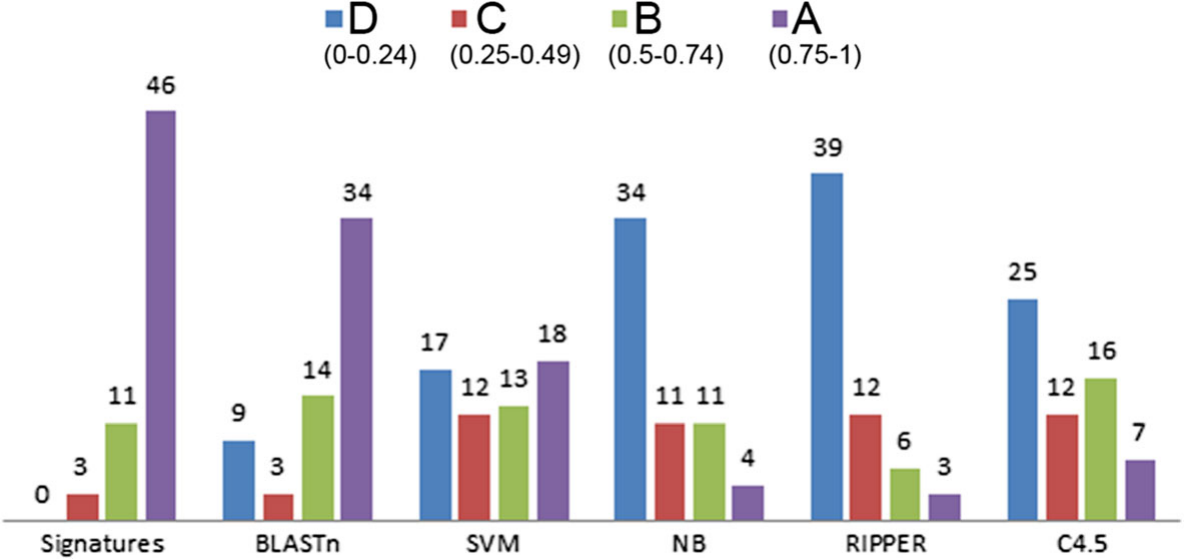
The comparative assessment of species signatures with BLASTn, SVM, Naive Bayesian, RIPPER, and C4.5 methods using F-measure values (**a**) from 0.75 to 1 (**b**) from 0.5 to 0.74 (**c**) from 0.25 to 0.49, and (**d**) from 0 to 0.24

We categorized the performance of the signatures according to the F-measure (higher to lower) and assigned them into four main categories based on F-measure value interval namely A (0.75–1), B (0.50–0.74), C (0.25–0.49), and D (0–0.24). We displayed the classification accuracy of the 60 species signatures with all other methods (Fig. 4). The detailed distribution of each species with the recall and precision and F-measure values of all methods are presented in Additional file 6. In A category, we obtained 46, 34, 18, 4, 3, and 7 species out of the 60 species for signatures, BLASTn, SVM, NB, RIPPER, and C4.5 respectively. In the ‘B’ category, there were 13, 14, 13, 11, 6, and 7 species could show comparable F-measure value for signatures, BLASTn, SVM, NB, RIPPER, and C4.5 respectively. We noticed that only three species grouped into C and D categories for Signatures, whereas 12, 29, 42, 51, and 37 species are grouped in BLASTn, SVM, NB, RIPPER, and C4.5 respectively. These results demonstrate that the performance of other methods in this category is the worse than signatures. However, BLASTn performance has better F-measure, followed by SVM, NB, RIPPER, and C4.5 considering A and B categories. The C4.5 method performs best in category B, which classified 16 species in comparison with Signatures (13 species), BLASTn (14 species), SVM (13 species), NB (11 species), and RIPPER (6 species) methods. Overall, from this result, we observed that signatures are the best in terms of recall and precision followed by BLASTn, SVM, C4.5, NB and RIPPER methods.

### QR code signature based ‘matK-QR Classifier’ software

**Fig. 5:**
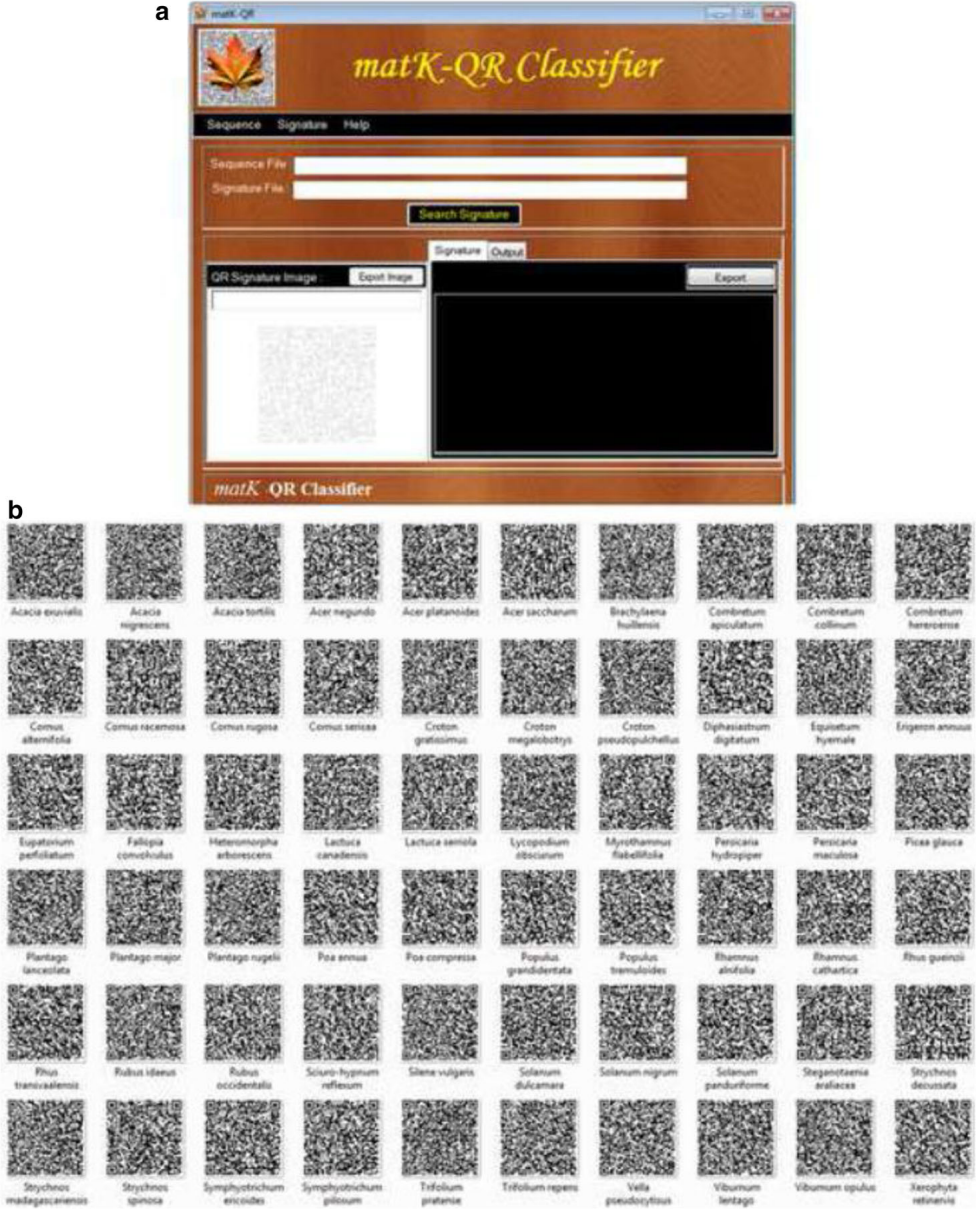
The matK-QR Classifier software (**a**) Graphical user interface (**b**) 60 species signatures encrypted into QR-codes

In order to show that the signatures are capable of identifying the correct plant species, we have developed and tested ‘*matK*-QR Classifier’ software with QR code symbology. The graphical interface and encrypted QR codes of the signatures are shown in Fig. 5. For species identification using signatures, the user may need to upload query *matK* sequences in FASTA format using the menu option (Sequence- open-Browse) (Fig. 5a). The user can choose the species QR signature of interest by using the option ‘Signature Menu-Open QR signature- Select QR code signature (ex. *Acacia exuvialis*.png)’ (Fig. 5b). Now the software decodes QR-code into the regular expression and proceeds further for searching it against query *matK* sequences. Finally, ‘Search’ option performs the species identification task by searching for the signature in the query sequence. The species name of the matched signature is assigned to the query sequence. After clicking on ‘show result’, the identified and non-identified species sequences summary is returned to the result panel. The output file contains information on sequence length, the location of signature in sequences and results can export in text files. Also, there is an option to convert FASTA sequence into the QR code and vice-versa under the ‘Signature menu-Generate QR codes’. The step-by-step analysis procedure is mentioned in the user manual to perform species identification using QR signatures (Additional file 7). The *matK*-QR Classifier, QR Signatures, and sample sequences file can be obtained from http://www.neeri.res.in/matk_classifier/index.htm.

## Discussion

In the present study, we showed the applicability of signature-based identification of plant species by using *matK* gene sequences. For the signature generation, the marker gene selection is an important consideration and it should hold enough variation to classify the species [29]. Our results revealed that *matK* gene showed greater discrimination success as compared to the *rbcL* gene (Table 1). Based on species percentage discrimination, we confirmed that the *matK* gene could be the best target for the signature generation due to the presence of variable nucleotide sites. As a consequence, we generated 60 species signatures to classify closely related sequences by using the PSSM information (Additional file 5). These results are consistent with earlier plant DNA barcoding studies that mentioned *matK* as a suitable barcode; this gene can discriminate morphologically similar and closely related species [13, 20, 30, 31].

The signature generation is a multistep procedure which depends on data set selection, alignment, PSSM nucleotide conservation, discriminating patterns identification and distance calculation among sequential patterns (Fig. 1). The generated species signatures showed that *matK* gene contains species-specific distinguishing patterns separated by a precise distance which can be considered as a regular expression (Fig. 2). In most cases, we noticed that a single nucleotide substitution was responsible for discrimination instead of larger patterns across species in the same genus (Fig. 3). This is due to the fact that the *matK* gene is highly conserved with low evolutionary distance rate [32].

In Weitschek et al. [19], the results indicated that the SVM and Naïve Bayes method performs the best than the RIPPER and C4.5 methods mainly on the data sets. In the present study, we showed the signature perform better than supervised methods (SVM and Naïve Bayes) specific to *matK* DNA barcodes of targeted plant taxa. This result supports an earlier finding by Seo TK [33] study that SVM method poorly performed in sequence classification for amphibians and dipterans due to the reduction in time durations of the ancestral populations during the evolutionary processes. In the case of species identification of insect (class Insecta) with COI gene sequences, the naïve Bayesian classifier method was used and showed better results [34]. In contrast, we obtained poor performance of the naïve Bayesian method as compared to signatures methods for targeted plant species classification. In previous studies, VIP Barcoding and BLOG tools were developed based on CV and K2P nearest-neighbour, and character-based methods to analyze DNA barcodes sequences [15, 35]. On similar approach, we used the discriminative nucleotide pattern to classify DNA barcodes using MSA and PSSM method. However, we generate *matK* gene-specific signatures for plant species identification. As stated above in the results section, the majority of the species are correctly classified by signatures method in category A with a high level of F-measure confidence. A signature method obtains superior classification performances with respect to all other methods. This finding confirms the fact that these signatures are more reliable for corresponding species identification. Although accuracy is low in the ‘B’ category, we find a high level of congruence between the signatures with all other methods. The possible reason behind this is that few bp variations exist in the *matK* gene sequence; due to this, all methods could not provide correct species identification. Signatures performed well in C and D categories to classify sequence with respective species, which underlies the importance of pattern based approach. Though signatures performed well in sequence classification, there could be probable limitations of signatures. 16 out of 60 species signatures produced false positive predictions (matched with other species sequences). It may be speculated that the possible reasons for this observation are partial sequence length, ambiguous nucleotide position, and the mismatch of the nucleotide distances between the two patterns. We acknowledge that signatures are not suitable to test on simulated data set as it was generated on the real DNA barcode dataset available in public databases.

For practical application of signatures, we developed the user-friendly graphical ‘*matK*-QR Classifier’ software on the Windows platform for signature based species identification (Fig. 5). This software accepts *matK* gene sequences and uses the discriminating pattern information in the form of signatures to identify plant species. The software allows users to select the QR-coded signature for a species of interest out of 60 signatures (Fig. 5b). It performs regular expression search against user defined query *matK* sequence. *matK*-QR Classifier software has various advantages over other types of DNA barcoding methods. Firstly, the user doesn’t need to pre-process input barcode sequences, alignment, and parameters setting to run the analysis like WEKA supervised methods (SVM, NB, and others). Earlier, it was pointed out that the alignment-free approach mainly features vectors and rule-based methods take low computing processing power [36, 37], due to this reason a signatures-based method is faster than the other methods because of regular expression based search. Secondly, *matK*-QR Classifier delivers ease-of-use, an easily accessible graphical user interface to the user. Third, *matK*-QR Classifier does not require pre-defined parameters and configuration prior to signature search against sequences. In contrast, several steps are required to be performed for other methods with different parameters, which are rather computationally expensive in case of a large number of sequences [38, 39]. Lastly, there is no requirement of training dataset or reference knowledge to perform analysis, which is one of the limitations of supervised methods like SVM and the Naïve Bayes [19].

Taking all these observations into account, our study suggests that PSSM method can be helpful to classify closely related species by considering large numbers of the *matK* sequences as compared to *rbcL* sequences. This outcome of study leads to generate signatures by using the *matK* sequences. To our knowledge, *matK*-QR Classifier is the only signature-based software to identify plant species based on *matK* gene-specific discriminating patterns. *matK* Classifier software addresses an important need of users, a graphic user interface providing a simple FASTA format sequence input and QR code signatures. We propose that signatures-based *matK*-QR Classifier can useful software for facilitating the plant species identification along with existing DNA barcoding methods and may also be applicable as a species identification tool for other organisms. In conclusion, we suggest that a similar approach could generate the signatures with single or multiple loci for plant species identification that is important in biodiversity analysis and routine identification purpose in plant evolutionary studies.

## Additional files

Additional file 1: The details of CBOL working group plant species dataset (*matK* and *rbcL* loci) used in the present study. (XLSX 29 kb)
Additional file 2: The PSSM and distance matrices of *matK* and *rbcL* loci datasets. (XLSX 2899 kb)
Additional file 3: The nucleotide variable sites identify at genus level using the position probability matrix of PSSM. (XLSX 17 kb)
Additional file 4: Discriminating and not discriminating species list obtained through PSSM species discrimination analysis. (XLSX 381 kb)
Additional file 5: The *matK* gene-specific nucleotide pattern-based signatures for plant species. (DOCX 26 kb)
Additional file 6: The details of the recall, precision and F-measure values of signatures, BLASTn, SVM, Naive Bayesian, RIPPER, and C4.5 methods. (XLSX 14 kb)
Additional file 7: The user manual of *matK*-QR Classifier software. (PDF 716 kb)

## Abbreviations

BLAST: Basic local alignment search tool
Bp: Base pair
CBOL: The consortium for the barcode of life
matK: MaturaseK
MSA: Multiple sequence alignment.
PSSM: Position specific scoring matrix
QR: Quick response
rbcL: Ribulose-1, 5-bisphosphate carboxylase
