# Supplementary material for: matK-QR classifier: a patterns based approach for plant species identification
Source: BioData Min. 2016 Dec 9;9:39. doi: 10.1186/s13040-016-0120-6 (PMC5148893; doi:10.1186/s13040-016-0120-6)
Supplement: Additional file 5: — The matK gene-specific nucleotide pattern-based signatures for plant species. (DOCX 26 kb) [file 13040_2016_120_MOESM5_ESM.docx]

Additional file 5: The *matK* gene specific species signatures.

| **No.** | **Species name** | **Signature** | |
| --- | --- | --- | --- |
|  | *Rhus transvaalensis* | GCCTCTTCTT\w{256}T\w{3}G\w{32}T\w{29}A\w{19}TTT\w{74}A\w{33}GC\w{4}C\w{30,36}CC\w{24}G\w{31}C\w{8}G\w{138}CGATTT | |
|  | *Rhus gueinzii* | T\w{3}G\w{32}T\w{29}A\w{19}TCT\w{74}A\w{33}GC\w{4}C\w{30,36}CC\w{24}G\w{31}C\w{8}G\w{132}ATTGACC | |
|  | *Diphasiastrum digitatum* | | TCATTGCTG\w{100,108}GATAAATGATTTA |
|  | *Heteromorpha arborescens* | TTTTTGCTAAGGC\w{36}C\w{50}G\w{67}C\w{195}G\w{8}TAACGTATTGGGGCA\w{49}GGCCTA\w{18}AATATTT | |
|  | *Sciuro-hypnum reflexum* | AGTTACTTCT\w{71}T\w{5}A\w{3}TT\w{7}A\w{2}AA\w{3}G\w{2}GA\w{0,3}G\w{19,23}T\w{0,2}T\w{8,12}T\w{10,14}A\w{5,9}G\w{0,2}T\w{0,2}G\w{1,5}T | |
|  | *Steganotaenia araliacea* | TTGCATTTATTACGA\w{118}C\w{37}C\w{215}A\w{94}GCGGCGAAACCCTTC | |
|  | *Vella pseudocytisus* | TATGAGTA\w{356}ATTTTTCCGTATGGTTTCAATCGCAA\w{15}C\w{11}G\w{18}G\w{21}A\w{20}T\w{8}A\w{24}G\w{70}T\w{13}A\w{13}G\w{8}A\w{1}A\w{5}A\w{2}A\w{3}TC\w{59}C\w{23}GAATAAAATATATA | |
|  | *Xerophyta retinervis* | T\w{4}G\w{25}G\w{36}A\w{4}G\w{198}T\w{18}T\w{12}CT\w{74}C\w{14}C\w{30}C\w{8,12}G\w{7,11}T\w{27}CCTATT | |
|  | *Erigeron annuus* | AATACA\w{138,142}GGAAAAAGAGAGCACTTTCCCAGGGCTT\w{5}G\w{51}G\w{6}C\w{229}A\w{6}TGATACTCTTGTTCCAATTATGCCTCTGATTGGATCA | |
|  | *Plantago lanceolata* | GTCTTTCTTAAGGTT\w{12}G\w{20}G\w{10}G\w{46}CGTCTCTTTTTAT\w{26}C\w{33}T\w{11,15}C\w{21,25}T\w{4,8}A\w{1,5}G\w{5,9}C\w{6,10}C\w{0,3}C\w{11,15}G\w{17,21}T\w{37,41}G\w{14,18}T\w{26}AAGCCCAA | |
|  | *Plantago major* | GTCCTTCTTAAGGTTAT\w{10}G\w{20}G\w{10}G\w{46}CGTCTCTTTTTAT\w{26}C\w{33}T\w{11,15}C\w{15}CAA\w{3,7}T\w{4,8}A\w{1,5}G\w{5,9}C\w{6,10}C\w{0,3}C\w{11,15}G\w{17,21}T\w{37,41}G\w{14,18}T | |
|  | *Plantago rugelii* | CCCTTTTT\w{158}CTCTAAATAA\w{175}GTCCTTCTTAAGGTTAC\w{10}G\w{20}G\w{10}G\w{46}CGTCTCTTTTTAT\w{26}C\w{33}T\w{11,15}C\w{21,25}T\w{4,8}A\w{1,5}G\w{5,9}C\w{6,10}C\w{0,3}C\w{11,15}G\w{17,21}T\w{37,41}G\w{14,18}T | |
|  | *Brachylaena huillensis* | ATTAAGAT\w{13}GTGTCATAATTGGGATAGTCTTATTAC\w{35,40}GAAATCACAGACTATTCTTCTTCC\w{32}GGCTTCATCTTTC\w{4}TAACCAA\w{52}TCTATGGAAAAATAGAGCATCTTGCAGAA\w{14}TTTTCAAGCTAATTTATGGT\w{46}AATTCTTGC\w{93}GATTTATATA\w{50}[GA][CG][GC]G[CG]TAAAGCCTTCAA\w{24,28}GCATTTCTAATCGA | |
|  | *Combretum apiculatum* | TTTGAACGAA\w{13}AAAAATCGAACAT[TC]TT\w{30}C\w{10}GG[GA]TTT\w{18}G\w{3}C\w{19}TATTTTG\w{21}A\w{3}G\w{86}TAGGTAAGTA\w{149}T\w{14}GTAACG[CT]ATT | |
|  | *Combretum hereroense* | TTTGAACGAA\w{13}AAAAATCGAACAT[TC]TT\w{30}C\w{10}GG[GA]TTT\w{18}G\w{3}C\w{47}A\w{3}G\w{241}TTGTTAAAA | |
|  | *Combretum collinum* | TTTGAACGAA\w{13}AAAAATCGAACAT[TC]TT\w{30}C\w{10}GG[GA]TTT\w{18}G\w{3}C\w{47}A\w{3}G\w{239}CATTGCTT | |
|  | *Cornus sericea* | AATAAAA\w{234}TTCTGGCTG\w{136}ATATCTTT\w{8}CGCCCAAACCCCT\w{49}GATTACGAAGTTCGA | |
|  | *Cornus alternifolia* | ATATGAA\w{190}TTCTGGCTG\w{136}ATATCTTT\w{8}CGCCCAAACCCCT\w{49}GATTACGAAGTTCGA | |
|  | *Cornus racemosa* | TTTCCTA\w{72}TTCTGGCTG\w{83}CCAGGAACGA\w{43}ATATCTTT\w{8}CGCCCAAACCCCTCAATGGTA\w{41}GATTACGAAGTTCGA\w{178}AAGAGTTT | |
|  | *Cornus rugosa* | TTCATAT\w{59}TTCTGGCTG\w{136}ATATCTTT\w{8}CGCCCAAACCCCT\w{49}GATTACGAAGTTCGA | |
|  | *Eupatorium perfoliatum* | TATACTTCTT\w{35,55}CTTCTCA\w{15,35}TTATTGAA\w{20,40}TCTTGCA\w{83,103}TCAAAAG\w{84}GGATTGATATA\w{11}CAATAATTC | |
|  | *Fallopia convolvulus* | TTTG\w{64}CCAATTT\w{25}TTATTG\w{35}C\w{4}T\w{49}G\w{50}G[GT]ATTTA\w{7}CTTT\w{23}GGACC[C]TTTTTT\w{267}GACCCTAA | |
|  | *Lycopodium obscurum* | CC\w{2}G\w{5}G\w{3}C\w{1}A\w{6}T\w{6}T\w{8}A\w{5}G\w{5}C\w{7}T\w{55}C\w{3}A\w{10}G\w{10}A\w{7}T\w{1}C\w{11}G\w{5}A\w{237}TCCAAAC | |
|  | *Myrothamnus flabellifolia* | GTATCGTAATTG\w{10}A\w{4}T\w{8}A\w{67}A\w{10}A\w{98}A\w{33}T\w{50}C\w{108}TATAAACCAATTATCCAATCATTCTCT\w{232}GGGCGGATAT | |
|  | *Populus grandidentata* | TACTAAGGATTGGG\w{7}CC\w{32}ATTCGAT\w{85}T\w{21}C\w{29}T\w{25}A\w{70,80}GGATAATATTATGAAT | |
|  | *Populus tremuloides* | TACTAAGGATTGGG\w{7}CC\w{37}A\w{62}TTATCTTG\w{16}T\w{21}C\w{29}T\w{25}A\w{70,80}GGATAATATTATGAAT | |
|  | *Rhamnus alnifolia* | TTTCTATTT\w{132}GAATCTATT\w{60}G\w{2}G\w{39}G\w{129}T\w{14}G\w{28}A\w{24}C\w{3}C\w{55}C\w{42}G\w{5}C\w{13}G\w{33}T\w{2}C | |
|  | *Rhamnus cathartica* | GAATATATT\w{60}G\w{2}G\w{39}G\w{129}T\w{14}G\w{28}A\w{24}C\w{3}C\w{55}C\w{42}G\w{5}C\w{13}G\w{33}T\w{2}C | |
|  | *Strychnos decussata* | ACAAATCCATTTTTGT\w{105}TAAGGATTA\w{9}A\w{79}C\w{224}A\w{26}T\w{69}GGATATTATTGA | |
|  | *Strychnos madagascariensis* | TCTTATTG\w{13}CAGTTT\w{10}ACAAAAAG\w{57}TTTCCTCTTT\w{101}TAAGGATTA\w{9}A\w{79}C\w{26}TTGTCAATT\w{189}A\w{26}T\w{69}GGATATTATTGA | |
|  | *Strychnos spinosa* | TAAGGATTA\w{9}A\w{79}C\w{26}TTGCCAATT\w{30}ACTCGGGA\w{151}A\w{26}T\w{69}GGATATTATTGA | |
|  | *Symphyotrichum ericoides* | GAAACATTACTTTGTCAAT\w{100}TGCGGTTAAAG\w{57}AAGGAGT | |
|  | *Symphyotrichum pilosum* | TTGGAATAG\w{207}TCCCGGG\w{110}GAAACATTACTTTGTCAAT | |
|  | *Acacia nigrescens* | TCATTTA\w{4}GGCTCTTT\w{38}AAAAAAAAGGA\w{123}CTGGAGTCTTTTTTGAA\w{26}ACATTTTGTGG\w{20}CCGTCCACCC\w{22}C\w{51}CCCTTTT\w{16}A\w{10}TCCATTTA\w{12}TTTTTTG\w{48}CATTTTAC\w{24,25}GGCTAAATCC\w{17}A\w{19}A\w{33}T\w{6}A\w{7}C\w{8}G\w{3}A\w{8}AGCAAAAT\w{39}GGGCCGATT | |
|  | *Acacia tortilis* | TTGGAGTCCTTTTTGAG\w{26}ATATTTTGTAG\w{20}CCGTCCACCC\w{22}C\w{51}C\w{20}A\w{10}TATCCATTTA\w{12}TTTTTTG\w{48}CATTTTAC\w{24,25}G\w{26}A\w{19}A\w{33}T\w{6}A\w{7}C\w{8}G\w{3}A\w{8}AGCAAAAT\w{89}AGAGATTTTTCT | |
|  | *Acacia exuvialis* | TTTTTTTCAA\w{108}TTGGAGTCCTTTTTGAG\w{26}ACATTTTGTAG\w{20}CCGTCCACCC\w{22}C\w{30}ATTCTCGCTT\w{11}C\w{20}A\w{10}TATCCATTTA\w{12}TTTTTTG\w{48}CATTTTAC\w{24,25}G\w{26}A\w{19}A\w{33}T\w{6}A\w{7}C\w{8}G\w{3}A\w{8}AGCAAAAT | |
|  | *Acer negundo* | GTATTTTAATTGGAATA\w{143}TTTTCAGGACTC\w{34}AAGATCT\w{15}TAATGATT\w{16}GTTGTTCAAGCATC\w{30}AATCCGTTC\w{55}TTTATGG\w{12}CGCGTGTG | |
|  | *Acer platanoides* | GTATTTTAATTGGAATA\w{143}TCTTCAGGACCC\w{34}AAGATCT\w{15}TAATGATT\w{16}GTTGTTCAAGCATC\w{30}AATCCGTTC\w{55}TTTATGG\w{12}CACGTGTG | |
|  | *Acer saccharum* | GTATTTTAATTTGAATA\w{40}AAGTAATCCA\w{45}TCAATCTT\w{17}AATCTTC\w{17}CTTCAGGACT\w{70}GACTACTTATGGTTGTTCAAGCAT\w{113}TCACGTGTG | |
|  | *Croton gratissimus* | GTCTTGGAATAGG\w{77}TTCTCATGT\w{50}TACGACCAAC\w{96}GGTCAAAGATCCTTTCC\w{54}TTCTGATGA\w{15}ACCTTGTCAAT\w{130}AAATACTAG\w{100}TAACGCAGT\w{87}TCATTACT | |
|  | *Croton megalobotrys* | GTATTGGAATGGG\w{77}TTCTCATGT\w{50}TACGATCAAC\w{96}GGTCAAAGATCCTTTCC\w{54}TTCTTATGA\w{15}ACCTTGTCGAT\w{130}AAATGCTAG\w{100}TAACACAGT\w{87}TCATTATT | |
|  | *Croton pseudopulchellus* | GTATTGGAATAGG\w{77}TTCTTATGT\w{50}TACGATCAAC\w{96}GTTCAAAGATCCCTTTC\w{54}TTCTGATGA\w{15}ACTTTGTCAAT\w{130}AAATGCTAG\w{100}TAACGCAGT\w{87}TCATTATT | |
|  | *Equisetum hyemale* | GCTAAAAAT\w{7}TTATTTAT\w{20}ATTCGTC\w{17}CTTTTATA\w{19}ATTCGCTTCC\w{22}AATTAAT\w{21}CATTAATT\w{32}CAGGTTCT\w{24}CAAAAACTAC\w{31}TTATTATTGTTTCA | |
|  | *Lactuca canadensis* | GAAAGCCAG\w{20}ATAACAG\w{10}TTCCTATA\w{27}CGGCTTCC\w{41}TCTGTAGC\w{36}TCATCTTGT\w{10}GCCAGGGCTTTT\w{23}AGATCCT\w{89}CAATTTC\w{15}TACCTGTGGT | |
|  | *Lactuca serriola* | GATTACTTCAA\w{38}CACAGACTATTC\w{5}CCTATA\w{27}CGGCTTCC\w{41}TCTGGAGCC\w{38}TCTTGCA\w{4}TCTTTGC\w{31}AAAGATC\w{65}ATGAATAA\w{22}TTCTGGAAATCT\w{9}CTGTGGT | |
|  | *Persicaria hydropiper* | AGAAAGAT\w{8}CAAAATCCGAATAAA\w{45}TCCATCTTCGTTT\w{12}AATCCTCTC\w{17}TACGGCGCCC\w{178}CTTTGTCAATC\w{18}CTCTATGGT\w{175}GGTTGGA\w{105}TGTAGAAACC | |
|  | *Persicaria maculosa* | TCCATCTTCGTTT\w{12}AATCTTCTC\w{17}TACGGCGCCC\w{178}CTTTGTCAATC\w{18}CTCTATGGT\w{175}GGTTGGA\w{105}TGTAGAAATCT | |
|  | *Picea glauca* | C\w{3}T\w{6}C\w{1}C\w{15}C\w{15}A\w{17}TC\w{14}C\w{14}A\w{8}C\w{52}C\w{12}A\w{15}C\w{2}T\w{5}C\w{9}TACTAATCT\w{7}AA | |
|  | *Poa annua* | TTTTTTTCAAC\w{13}G\w{15}G\w{8}T\w{15}A\w{4}G\w{15}C\w{11}G\w{4}C\w{18}G\w{4}C\w{12}C\w{1}AAAAAG\w{82}GTTACTGCCCTCATAGGATACTTAG | |
|  | *Poa compressa* | TTTTTTTCAAC\w{13}G\w{15}G\w{8}T\w{15}A\w{4}G\w{15}C\w{11}G\w{4}C\w{18}G\w{4}C\w{12}C\w{1}AAAAAG\w{82}CTTACTGCCCTCATAGGATACTTAG | |
|  | *Rubus idaeus* | CCAAAAAAATCT\w{51}GTATGTGAATAC\w{12}GTTTTTCT\w{36}TGGAATCT\w{53}TTTGCTAACTA\w{81}AGATACT\w{48}TGTCATT\w{86}CGACCGAAT\w{24}CTAGAAAA\w{40}TTAATTCC\w{15}AGGATCG\w{20}TAACACA\w{14}TAGTAA\w{9}GGCGGAT\w{44}AAATCTTT | |
|  | *Rubus occidentalis* | CAAAAAAATCG\w{70}TTTTACTTTTT\w{37}CTGGGATCT\w{58}TAACGATTTT\w{51}CGAGGAAAATCT\w{12}AAAGAGACTC\w{140}CGACGGAAT\w{23}GCTAGAAAA\w{108}TATTACTAAGT\w{57}GAAATATTTCT | |
|  | *Silene vulgaris* | TCTATTTATTGTTTTTTTAAA\w{14}ATTC[AT]TGTT\w{27}CGAATCTATTTTA\w{15}CAATCCTCTC\w{34}ACGCACTTTTTTCTAC\w{5}ATTA[AG]AAT\w{12}T\w{16}GC\w{1}G\w{11}C\w{16}C\w{3}C\w{12}A\w{19}GCCTCAA\w{20}C\w{81}G\w{8}C\w{17}T\w{24}C | |
|  | *Solanum dulcamara* | CTCCTTTTCAAAAAAAAATCAAAGA\w{2}CTTCTTTT\w{27}GCGAAT\w{86}AAAATA\w{44}ATGGTTATTCAAGGACC\w{43}TTCAAAAGGGACGTTT | |
|  | *Solanum nigrum* | CTCCTTTTCAAAAAAAACTCAAAGA\w{2}CTTCTTCT\w{27}GCGAAT\w{86}AAAATA\w{44}CTGGTTATTCAAGGACC\w{43}TTCAAAAGGGACGTTT | |
|  | *Solanum panduriforme* | CTCTTTTTCAAAAAAAAATCCAAAA\w{2}CTTCTTCT\w{27}GCGAAT\w{86}AAAATA\w{44}ATGGTTATTCAAGGATC\w{43}TTCAAAAGGGACGTTT\w{169}TGTTTGAAA | |
|  | *Trifolium pratense* | TCGTTTTATTACTA\w{5}A\w{9}C\w{30}C\w{20}TGTACGTGAAT\w{27}A\w{47}G\w{2}T\w{25}C\w{24}T\w{28}C\w{2}A\w{15}G\w{6}A\w{7}G\w{33}G\w{23}C\w{10}A\w{35}T\w{101}CATTTCT\w{50,70}TCGTTGG | |
|  | *Trifolium repens* | TTGTTTTATTCCCA\w{5}A\w{9}C\w{30}C\w{30}T\w{27}A\w{47}G\w{2}T\w{25}C\w{24}T\w{28}C\w{2}A\w{15}G\w{6}A\w{7}G\w{33}G\w{23}C\w{10}A\w{35}T\w{101}CATTTCT\w{50,70}TCGTTGG | |
|  | *Viburnum opulus* | AATTTGAAT\w{17}GAAAGCCAGC\w{25}GATTATTCTTCT\w{87}GAACCTTT\w{41}AGAAGTCTTTGCTAAGGCTTTTCA\w{26}T\w{213}GG\w{70}AATTATTTCTC\w{15}T\w{9}T\w{8}C\w{1}T\w{31}A\w{28}ACGATTTGGGCGTATATGCAGA\w{37}AAAGTT | |
|  | *Viburnum lentago* | GAAAGCCAGT\w{25}GATTATTCTTT\w{87}GGAACCTTT\w{41}CGAAGTCTTTGCTAAGGCTTTTCA\w{26}T\w{213}GA\w{70}AATTATTTCTC\w{15}T\w{9}T\w{8}[TC]\w{1}C\w{31}G\w{28}ACGATTTGGGCGTATATGCAGA\w{37}AAAGTT | |

^The signature (i.e. regular expression) matches subsequence from complete sequences that containing subsequent patterns with defined \w{n, m} distances. The \w stands for a word character (nucleotides) in regular expression. The distance between two patterns is {n, m}, where n is the minimum number of nucleotides and m is the maximum number; International Union of Pure and Applied Chemistry (IUPAC) coding has been referred to represent ambiguous base pairs mutation in pattern^
