# Supplementary material for: matK-QR classifier: a patterns based approach for plant species identification
Source: BioData Min. 2016 Dec 9;9:39. doi: 10.1186/s13040-016-0120-6 (PMC5148893; doi:10.1186/s13040-016-0120-6)
Supplement: Additional file 7: — The user manual of matK-QR Classifier software. (PDF 716 kb) [file 13040_2016_120_MOESM7_ESM.pdf]

matK-QR Classifier

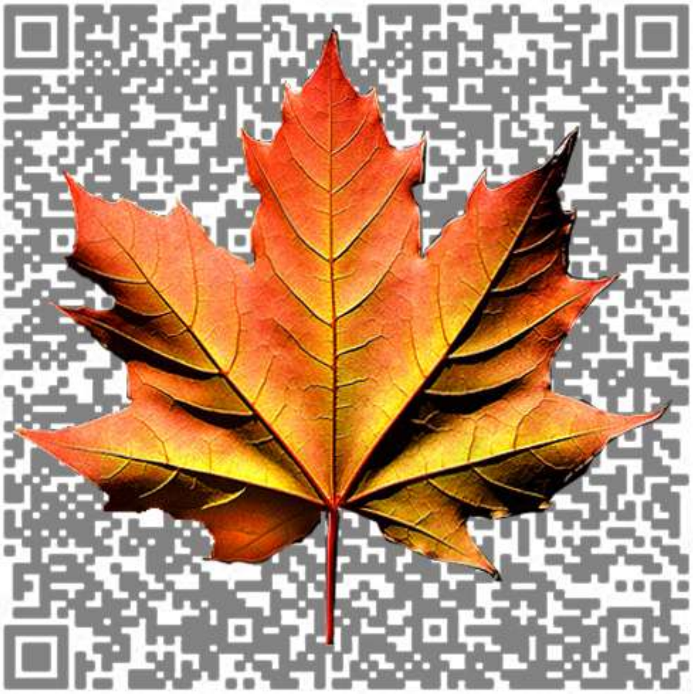

Contents:

1. About *matK*-QR Classifier software……………………………………….2

2. Contacts……………………………………………………………………..2

3. Availability…………………………………………………………………….3

4. Installation……………………………………………………………………..3

4.1. System Requirements

4.2. Steps to Installing *matK*-QR Classifier on Windows platform

5. An example of procedure to taxonomic identification…………………...5

1. About *matK*-QR Classifier software

***matK*-QR Classifier** software represent *matK* gene specific signatures as QR codes library in practical application for plant species identification.

2. Contact

Dr. Ravi P. More and Dr. Hemant J. Purohit

Department: Environmental Genomics Division

Institute: CSIR- National Envirnomental Engineering Reasearch Institute (NEERI)

Address: Nehru Marg, Nagpur – 440 015, Maharashtra, India

Tel: +917122249883

Fax: +917122243927

Email: hj_purohit@neeri.res.in

Technical Contact: ravimore7@yahoo.in

Website: www.neeri.res.in

3. Availability

The executable version of *matK*-QR Classifier and user manual are available under download section at website http://www.neeri.res.in/matk_classifier/matK_QR.htm. During downloading, If any massage (ex. matK-QR is not commonly downloaded) occurred, please select 'keep' option.

4. Installation

4.1. System Requirements

1. *matK*-QR Classifier was developed for use on Microsoft Windows® operating systems: Windows XP, 7 and 8 (32-bit) but it can install on Windows 7 (64 bit) platform also.
2. The computer on which *matK*-QR Classifier is installed should meet the following hardware requirements:
    - Windows 7 or 8 (32-bit/64 bit) is recommended.
    - The minimum computer requirements are at least 1 GB of RAM and 2 GB of available hard disk space with a Pentium® processor.
    - Microsoft .NET Framework 4 is required (Note: it will automatically install in internet connectivity)

4.2. Steps for installation *matK*-QR Classifier on Windows platform

i. Download software executable from URL mentioned above availability section.

ii. Unzip '*matK*-QR Classifier' Windows installation package using Winzip/Winrar software.

iii. After extraction, Double click on setup.exe file to launch and follow the setup wizard by clicking next button as shown in following Figure 1.

iv. After successful completion of setup, *matK*-QR Classifier icon will be appeared in Start Menu. Also, user can find at location: (C:\Program files\ *matK*-QR Classifier). Software shortcut icon will created on Desktop by default.

v. User can select either of the above option to open *matK*-QR Classifier software.

vi. If any error massage occurred during the uploading files, please provide administrator permission to access C:\ using following steps (Start-Control Panel-User account and family safety-User account-Change user account control settings-Never notify- OK- Restart Computer).

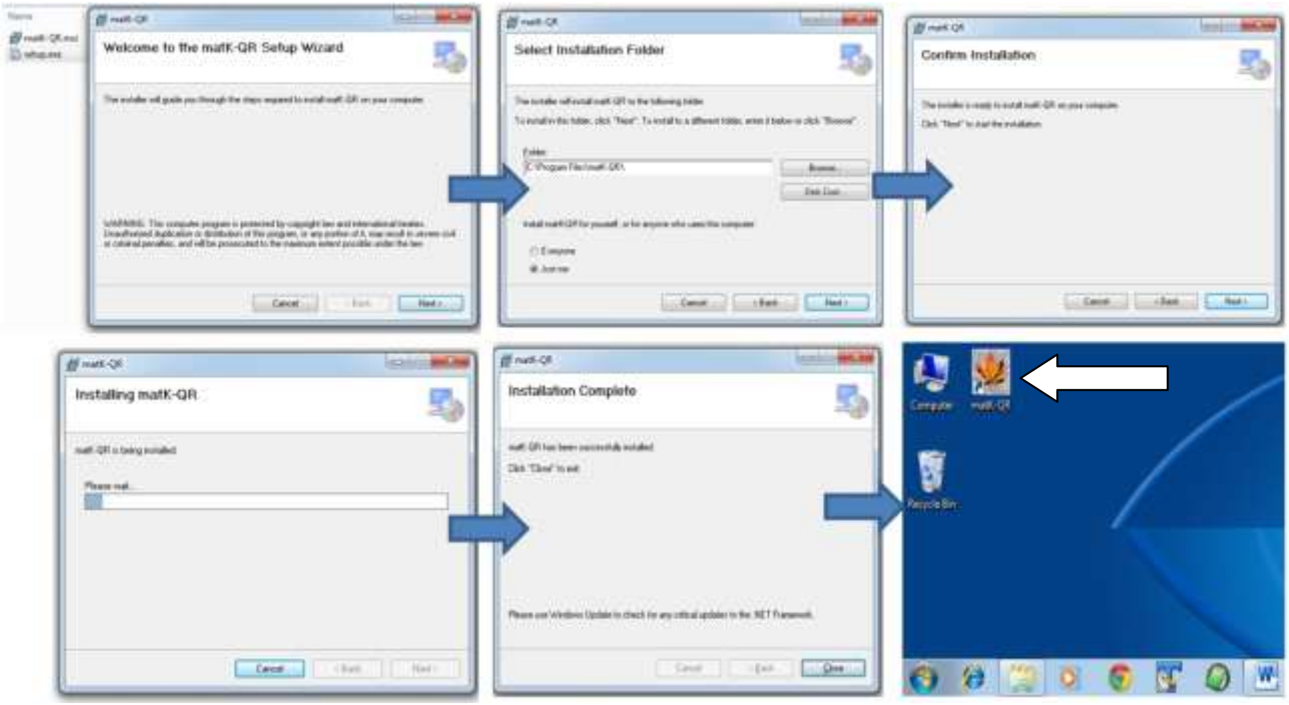

Figure 1: The screenshot of installation steps for *matK*-QR Classifier software.

An example of procedure to perform species identification through QR code encrypted Signatures:

The following are simple steps to perform taxonomic identification:

Step 1: Click on Sequence menu - Open Sequence file – choose either Sample Sequence File (Figure 2a) option or Browse file (Figure 2b) to input own query sequences.

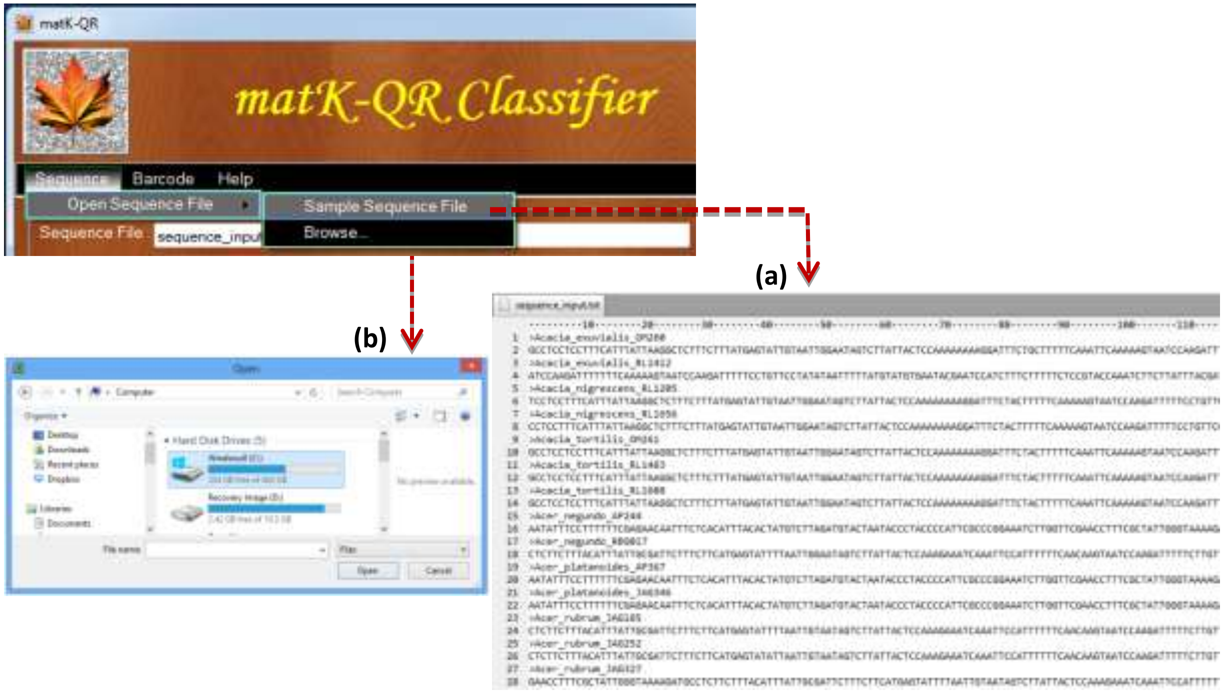

Figure 2: The screenshot of *matK*-QR classifier 'Sequence' menu.

Using this menu, user can select either 'Sample Sequence File' or upload *matK* sequences file from computer location using 'Browse' option as shown in Figure 2. Currently software accepts FASTA format *matK* sequences with .fasta/.txt/.fa/.seq/.fna extension file. Sample sequence file contains 345 studied sample *matK* sequences in this work.

Step 2: Click on Barcode Menu - Open QR DNA barcode – Sample QR DNA Barcode – Select Sample QR DNA Barcode (Figure 3a).

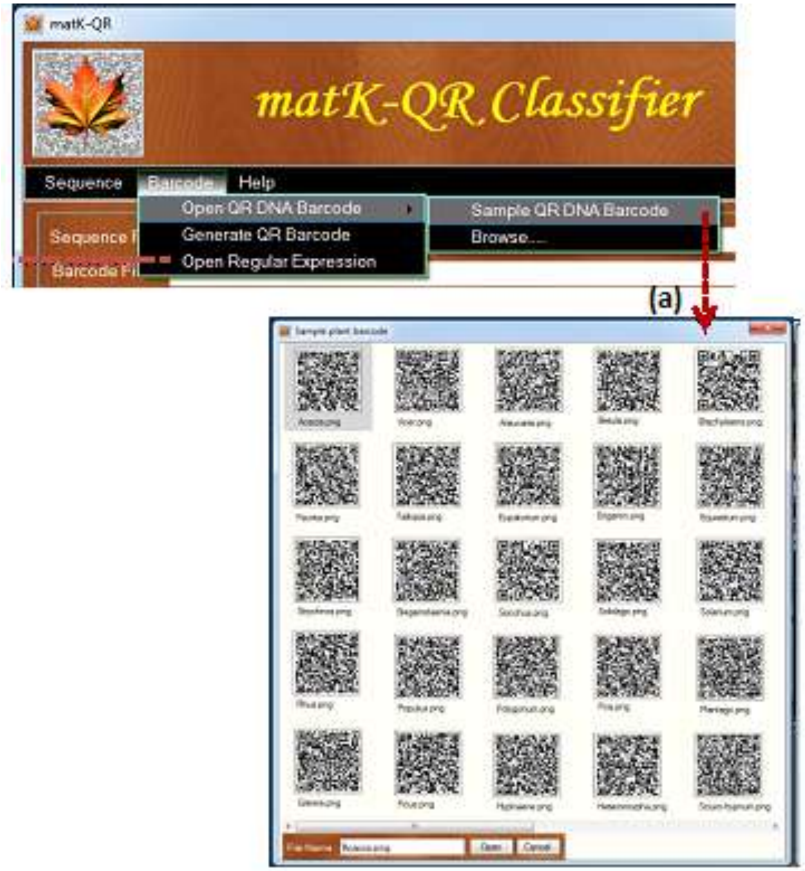

Figure 3: The screenshot of ‘Barcode menu’ window (a) QR-codes library (b) Regular expression

Using ‘Barcode’ menu, in ‘Open QR DNA Barcode’ option, user can select either single QR-code (Ex. Acacia.png as shown in Figure 3a).

Step 3**:** Click on ‘Search Barcodes’ and then click on ‘OK’ show result window (Figure 4).

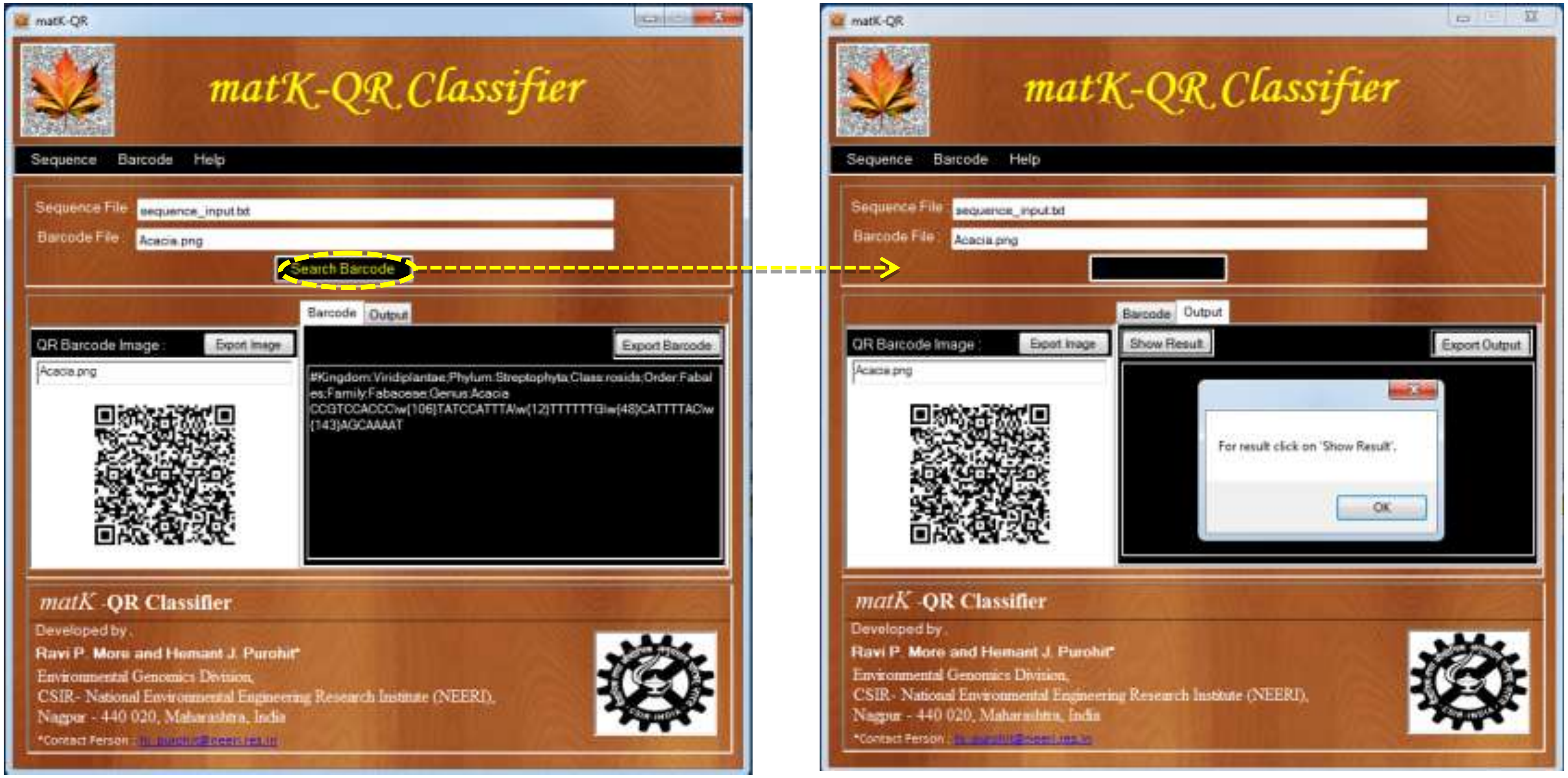

Figure 4: The screenshot of ‘Search Barcode’ window.

At this step, after click on ‘Search Barcode’ button (Figure 4), software perform regular expression based search of QR-code (signature) against *matK* sequences. Once analysis finished, click on OK of show result dialog box in order to view result summery.

Step 4: Click on ' Export Output' button and then choose desired folder to save output files (Figure 5).

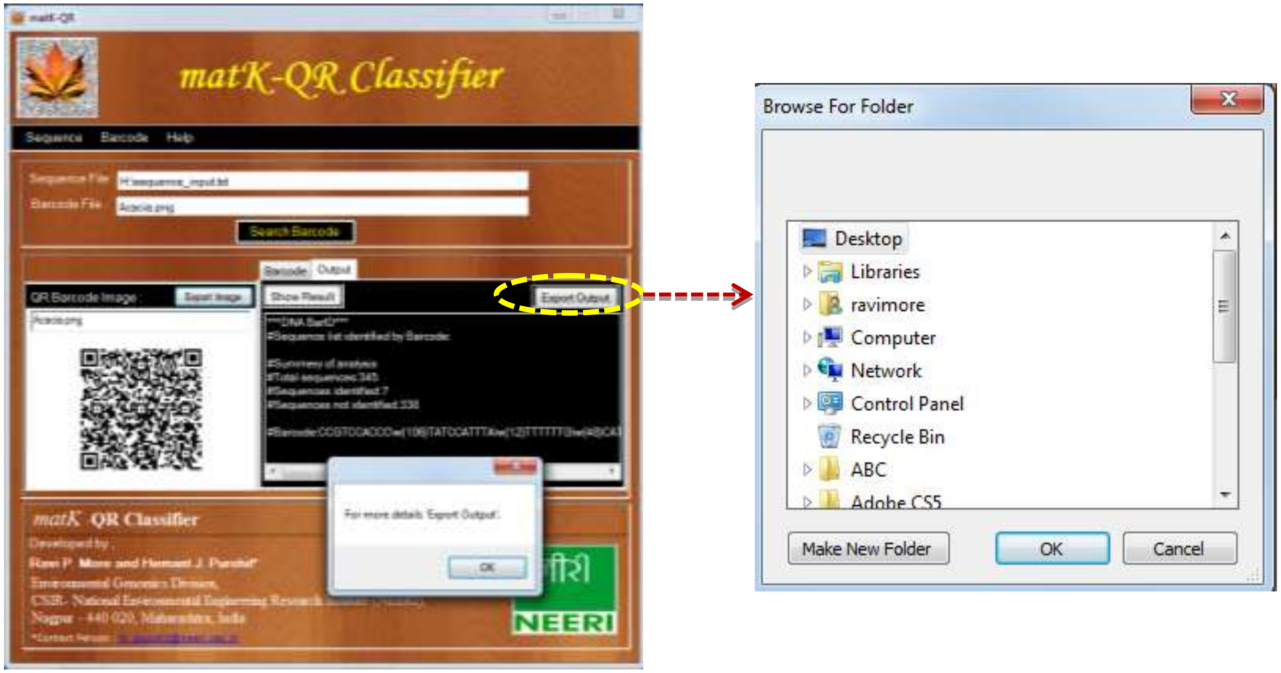

Figure 5: The screenshot of 'Export Output' window.

After click on 'Export Output' button, user can save output files in desired folder through 'Browse for folder' dialog as shown in Figure 5. Ex. Create 'Result' folder on Desktop to save files.

Step 5: Open result output files folder (Ex. Desktop/Result) and open output results files (Figure 6).

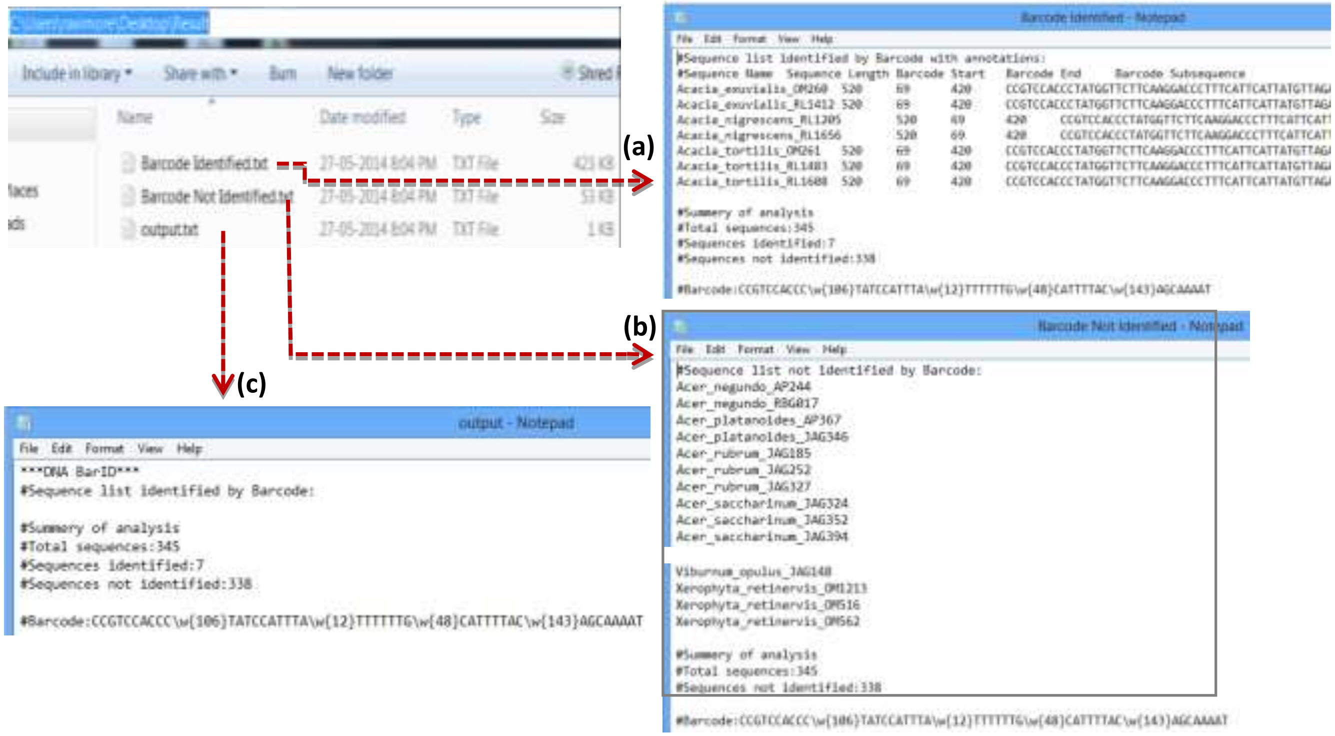

Figure 6: The screenshot of Results output file (a) Barcode Identified.txt (b) Barcode Not Identified.txt (c) output.txt.

Click on ‘Barcode Identified.txt’ tab-delimited file contains following fields: Sequence Name, Sequence Length, Barcode Start, Barcode End, and Barcode Subsequence (Figure 6a). In ‘Barcode Not Idetified.txt’ file provides not identified sequence name list (Figure 6b). The ‘output.txt’ file contains the total, identified, not identified sequences list and input Barcode (Figure 6c). User can export these data into the spreadsheet software like Microsoft Excel for making structured database.
